# High-dose intravenous selenium does not improve clinical outcomes in the critically ill: a systematic review and meta-analysis

**DOI:** 10.1186/s13054-016-1529-5

**Published:** 2016-10-28

**Authors:** William Manzanares, Margot Lemieux, Gunnar Elke, Pascal L. Langlois, Frank Bloos, Daren K. Heyland

**Affiliations:** 1 Department of Critical Care, Intensive Care Unit, Hospital de Clínicas (University Hospital), Faculty of Medicine, Universidad de la República (UdelaR), Avenida Italia, 14th Floor, Montevideo, 11.600 Uruguay; 2 Clinical Evaluation Research Unit, Kingston General Hospital, Kingston, ON Canada; 3 Department of Anesthesiology and Intensive Care Medicine, University Medical Center Schleswig-Holstein, Campus Kiel, Kiel, Germany; 4 Centre Hospitalier Universitaire de Sherbrooke, Hospital Fleurimont, Sherbrooke, QC Canada; 5 Department of Anesthesiology and Intensive Care Medicine, Jena University Hospital, Jena, Germany; 6 Department of Medicine, Queen’s University, Kingston, ON Canada

**Keywords:** Parenteral selenium, Intravenous selenium, Antioxidant micronutrient, Critically ill

## Abstract

**Background:**

Selenium (Se) is an essential trace element with antioxidant, anti-inflammatory, and immunomodulatory effects. So far, several randomized clinical trials (RCTs) have demonstrated that parenteral Se may improve clinical outcomes in intensive care unit (ICU) patients. Since publication of our previous systematic review and meta-analysis on antioxidants in the ICU, reports of several trials have been published, including the largest RCT on Se therapy. The purpose of the present systematic review was to update our previous data on intravenous (IV) Se in the critically ill.

**Methods:**

We searched MEDLINE, Embase, and the Cochrane Central Register of Controlled Trials. We included RCTs with parallel groups comparing parenteral Se as single or combined therapy with placebo. Potential trials were evaluated according to specific eligibility criteria, and two reviewers abstracted data from original trials in duplicate independently. Overall mortality was the primary outcome; secondary outcomes were infections, ICU length of stay (LOS), hospital LOS, ventilator days, and new renal dysfunction.

**Results:**

A total of 21 RCTs met our inclusion criteria. When the data from these trials were aggregated, IV Se had no effect on mortality (risk ratio [RR] 0.98, 95 % CI 0.90–1.08, *P* = 0.72, heterogeneity *I* ^2^ = 0 %). In addition, when the results of ten trials in which researchers reported on infections were statistically aggregated, there was no significant treatment effect of parenteral Se (RR 0.95, 95 % CI 0.88–1.02, *P* = 0.15, *I* ^2^ = 0 %). There was no positive or negative effect of Se therapy on ICU and hospital LOS, renal function, or ventilator days.

**Conclusions:**

In critically ill patients, IV Se as monotherapy does not improve clinical outcomes.

**Electronic supplementary material:**

The online version of this article (doi:10.1186/s13054-016-1529-5) contains supplementary material, which is available to authorized users.

## Background

Selenium (Se) is an essential trace element with anti-inflammatory and immunomodulatory properties, and it is currently considered the cornerstone of the antioxidant defense system [1, 2]. Over the past 30 years, a large number of basic and clinical studies have revealed the crucial role of Se in the maintenance of immune, metabolic, endocrine, and cellular homeostasis, which is attributed to its presence in selenoproteins, as the 21st amino acid selenocysteine [3]. These selenoenzymes are involved in redox signaling, antioxidant defense, thyroid hormone metabolism, and immune responses [4]. Thus far, critical illness with systemic inflammation and multiple organ dysfunction syndrome was deemed to be associated with an early reduction in plasma/serum Se and glutathione peroxidase (GPx) activity, where both parameters correlate inversely with the severity of illness and clinical outcome [5, 6].

Over the last two decades, researchers in several randomized clinical trials (RCTs) have evaluated the role of parenteral inorganic selenocompounds such as sodium selenite or selenious acid, either as a single-agent strategy or in combination with other antioxidant micronutrients (antioxidant cocktails) and using different dose regimens in critically ill patients with systemic inflammation. These studies have shown beneficial results in terms of reduction of infections, mortality, and other relevant clinical outcomes in the critically ill.

In 2005, the authors of the first comprehensive systematic review and meta-analysis on antioxidant nutrients in the critically ill [7] demonstrated that Se supplementation could be associated with a reduction in mortality, while nonselenium antioxidants had no effect on mortality. More recently, authors of other systematic reviews and meta-analyses [8–14] on Se therapy in intensive care unit (ICU) patients found that pharmaconutrition with parenteral Se monotherapy may be able to significantly reduce mortality in patients with sepsis, particularly when an intravenous (IV) bolus was provided and daily doses higher than 500 μg were administered. In addition, Se substitution was more effective in those patients with higher risk of death [13].

Nonetheless, since 2013, several studies of the effects of parenteral Se supplementation as single or combined therapy have been published [7, 14–17]. The REducing Deaths due to Oxidative Stress (REDOXS) trial [16] investigators were unable to find a therapeutic benefit of a combined Se supplementation regimen (300 μg enteral plus 500 μg parenteral). The most recent and largest RCT on Se monotherapy in severe sepsis and septic shock, the Sodium Selenite and Procalcitonin Guided Antimicrobial Therapy in Severe Sepsis (SISPCT) study [17], further demonstrated that high-dose IV sodium selenite was not associated with improved survival. Therefore, with the aim of elucidating the overall efficacy of parenteral Se as single or combined therapy (antioxidant cocktails) in adult critically ill patients, we performed an update of our previous systematic review and meta-analysis of the literature.

## Methods

### Study identification

We conducted a systematic review of the literature published between 1980 and 2015 using the computerized databases of MEDLINE, Embase, the Cochrane Controlled Trials Register, and the Cochrane Database of Systematic Reviews. Text words or MeSH headings containing “randomized,” “blind,” “clinical trial,” “parenteral,” “intravenous,” “selenium,” “sodium selenite,” “selenious acid,” “antioxidant cocktails,” “critical illness,” and “critically ill” were used without any language restriction. We also reviewed our personal files and comprehensive reviews for additional original studies.

### Study selection criteria

Original studies were included if they met the following criteria:

1. Randomized controlled trial study design with a parallel group
2. A population of critically ill adult patients (>18 years old), defined as patients admitted to an ICU (If the study population was unclear, we considered a mortality rate higher than 5 % in the control group to be consistent with critical illness.)
3. The administration of parenteral Se in the intervention arm (either with or without an initial bolus) as a single-agent strategy or in combination with other antioxidant micronutrients compared with a control group with a placebo
4. The evaluation of clinically relevant outcomes such as mortality, infectious complications, ICU or hospital length of stay (LOS), length of mechanical ventilation (MV), and new renal dysfunction including the requirement of renal replacement therapy

Clinical studies that reported only biochemical, metabolic, or immunologic results were excluded.

All original studies were evaluated and abstracted in duplicate independently by two reviewers using a data abstraction form that had been used previously [18]. A discussion was held and consensus was obtained between the reviewers when a disagreement occurred. When additional data were needed, we attempted to contact the authors of the published article. We scored the methodological quality of the original trials, aiming to obtain a score between 0 and 14, with the following high-quality criteria: (1) the extent to which randomization was concealed, (2) intention-to-treat (ITT)-based analysis, (3) extent of blinding, (4) baseline comparability of groups, (5) extent of follow-up, (6) description of treatment protocols and cointerventions in both arms, and (7) definition of clinical outcomes [18].

We designated studies as level I if all of the following criteria were fulfilled: concealed randomization, blinded outcome adjudication, and an ITT analysis, which are the strongest methodological tools to reduce bias. A study was considered as level II if any one of the above-described characteristics was unfulfilled.

### Data analysis

The primary outcome was overall mortality. Hospital mortality, when available, was used for the statistical analysis. If not reported, we used ICU mortality or 28-day mortality. When not specified, mortality was assumed to be hospital mortality. Secondary outcomes included infections, hospital and ICU LOS, MV days, and new renal dysfunction as defined by the authors of the original articles. We used the definition of infections employed by each author. The data from all trials reporting the specific outcome were combined to calculate the pooled risk ratio (RR) for mortality and infections, and pooled weighted mean difference (WMD) for LOS, both with 95 % CIs. All analyses were conducted using Review Manager (RevMan) 5.3 software, except for the test for asymmetry. Pooled RRs were calculated using the Mantel-Haenszel estimator, and WMDs were estimated by the inverse variance approach. The random effects model of DerSimonian and Laird [19] was used to estimate variances for the Mantel-Haenszel and inverse variance estimators. When possible, studies were aggregated on an ITT basis. Heterogeneity in the data was tested by a weighted Mantel-Haenszel chi-square test and quantified by using the *I* ^2^ statistics implemented in RevMan 5.3 software. Differences between subgroups were analyzed using the test of subgroup differences described by Deeks et al. [20], and the results were expressed using the *P* values. Funnel plots were generated to assess the possibility of publication bias, and the Egger regression test was used to measure funnel plot asymmetry [21]. Asymmetry was calculated using Comprehensive Meta-Analysis 3.0 statistical software (Biostat Inc., Englewood, NJ, USA). *P* values <0.05 and <0.10 were considered as statistically significant and indicators of a trend, respectively.

### A priori hypothesis testing

Significant differences in the protocols of the original studies were expected. Thus, several prespecified hypothesis-generating subgroup analyses were performed to identify potentially more beneficial treatment strategies. First, we compared the results of trials in which investigators administered parenteral Se as monotherapy with studies in which researchers provided parenteral Se in antioxidant cocktails. Based on previous RCTs showing a beneficial effect of an initial loading dose, those RCTs using an initial loading dose as an IV bolus of Se were then compared with trials those that did not. In addition, because researchers in previous trials found that daily doses higher than 500 μg were associated with better outcomes, we compared the results between three subgroups having different daily doses: lower than 500 μg, equal to 500 μg, and greater than 500 μg. Moreover, on the basis of a possibly larger treatment effect in patients with higher risk of death, we compared studies including patients with higher mortality vs. those with lower mortality. Mortality was considered to be high or low based on whether it was greater or less than the mean control group mortality of all the trials. Additionally, we postulated that trials with lower quality (level II studies) might demonstrate a greater treatment effect than those trials with higher quality (level I studies). Furthermore, as current evidence showed benefits in terms of reduction in mortality in septic patients, the results of RCTs performed only with patients with sepsis were compared with RCTs performed with heterogeneous patient populations (nonsepsis studies). We also assessed the effect of Se in soils according to the geographical region where the trial was conducted. For this purpose, we compared RCTs performed in deprived regions (Europe, South America, and Asia) versus trials performed in nondeprived regions (North America). Finally, given the interaction between Se and procalcitonin (PCT) in the SISPCT study [17], we conducted a sensitivity analysis excluding the PCT guidance group of patients.

## Results

### Study identification and selection

A total of 41 relevant citations were identified in the search of computerized bibliographic databases and a review of reference lists in related articles. Of these, we excluded 20 for the following reasons: 8 trials did not include ICU patients (mostly surgery patients) [22–29]; 1 study did not evaluate clinical outcomes [30]; 1 study compared high-dose with low-dose Se [31]; 3 articles were duplicates [32–34]; 4 articles were systematic reviews; 1 trial was published as an abstract [35], and we were unable to obtain the data from the authors to complete our data abstraction process; 1 study was not an RCT [36]; and in 1 trial Se was not given intravenously [37].

Ultimately, 21 studies [14–17, 38–54] met our inclusion criteria and were included; they comprised a total of 4044 patients (Tables 1 and 2). The reviewers reached 100 % agreement for the inclusion of the trials. The mean methodological score of all trials was 9 of a maximum possible score of 14 (range 4–13). Randomization was concealed in 9 (43 %) of 21 trials; ITT analysis was performed in 14 (67 %) of 21 trials; and double-blinding was done in 7 (33 %) of 21 of the studies. There were 6 level I studies and 15 level II studies. The details of the methodological quality of the individual trials are shown in Table 1.

**Table 1:** Randomized clinical trials evaluating selenium supplementation in critically ill patients

Study	Population	Methodology (score)	Intervention
Kuklinski 1991 [38]	Patients with acute pancreatic necrosis	C.Random: not sure	PN + selenium supplementation (500 μg/day) vs. PN without selenium supplementation
	*n* = 17	ITT: no	
		Blinding: no	
		(4)	
Zimmerman 1997 [39]	Patients with SIRS, sepsis, APACHE II score >15, and multiorgan failure score >6	C.Random: no	IV selenium as sodium selenite 1000 μg as a bolus and then 1000 μg sodium selenite 24 h as a continuous infusion over 28 days vs. standard
	*n* = 40	ITT: yes	
		Blinding: no	
		(6)	
Berger 1998 [40]	Burns >30 % TBSA	C.Random: yes	IV copper (40.4 μmol), selenium (159 μg), zinc (406 μmol) + standard trace elements vs. standard trace elements (copper 20 μmol, selenium 32 μg, zinc 100 μmol) from days 0 to 8, all received early EN
	*n* = 20	ITT: yes	
		Blinding: double blind	
		(12)	
Angstwurm 1999 [41]	Patients with SIRS and sepsis at 11 ICUs	C.Random: not sure	PN with high-dose selenium (535 μg × 3 days, 285 μg × 3 days, 155 μg × 3 days, and 35 μg thereafter) vs. low-dose selenium (35 μg/day for duration of study)
	*n* = 42	ITT: yes	
		Blinding: no	
		(10)	
Porter 1999 [42]	Surgical ICU penetrating trauma patients with Injury Severity Score ≥25	C.Random: yes	50 μg selenium IV every 6 h + 400 IU vitamin E, 100 mg vitamin C every 8 h, and 8 g of *N*-acetylcysteine every 6 h via nasogastric or oral route from days 0 to 7 vs. none
	*n* = 18	ITT: yes	
		Blinding: no	
		(9)	
Berger 2001 [43]	Trauma patients, surgical ICU	C.Random: yes	IV selenium supplementation (500 μg/day) vs. placebo (selenium group randomized further to two groups: 500 μg selenium alone vs. 500 μg selenium + 150 mg α-tocopherol + 13 mg zinc) given slowly for first 5 days after injury (all groups received EN)
	*n* = 31	ITT: no	
		Blinding: double	
		(9)	
Lindner 2004 [44]	Patients with acute pancreatitis admitted to the ICU	C.Random: not sure	IV sodium selenite dose of 2000 μg on day 1, 1000 μg on days 2–5, and 300 μg from day 6 until discharge vs. placebo (isotonic 0.9 % IV NaCl solution)
	*n* = 70	ITT: no	
		Blinding: single	
		(9)	
Angstwurm 2007 [45]	Multicenter mixed ICUs	C.Random: not sure	1000 μg selenium IV within 1 h followed by 1000 μg selenium for 14 days vs. NaCl (0.9 %) (all patients received EN or PN)
	*n* = 249	ITT: no	
		Blinding: double	
		(8)	
Berger 2007 [46]	Burns >20 % TBSA	C.Random: not sure	IV 100 ml of copper (59 μmol) + selenium (375 μg + zinc (574 μmol) vs. NaCl (0.9 %) from admission for 5–15 days
	*n* = 21	ITT: yes	Both groups were on EN
		Blinding: no	
		(8)	
Forceville 2007 [47]	Septic shock patients	C.Random: not sure	4000 μg selenium IV on day 1 followed by 1000 μg selenium for 9 days vs. NaCl (0.9 %) (all patients received EN or PN)
	*n* = 60	ITT: no	
		Blinding: double	
		(8)	
Mishra 2007 [48]	Septic ICU patients	C.Random: not sure	474 μg selenium IV × 3 days followed by 316 μg × 3 days, 158 μg × 3 days, and 31.6 μg thereafter vs. 31.6 μg selenium (all patients received EN or PN)
	*n* = 40	ITT: yes	
		Blinding: double	
		(9)	
Berger 2008 [49]	Mixed ICU	C.Random: not sure	IV selenium supplementation loading dose 540 μg/day + zinc (60 mg) + vitamin C 2700 mg + vitamin B 305 mg + vitamin E enteral 600 mg + vitamin E 12.8 mg IV for 2 days followed by half the dose of all vs. standard vitamins
	*n* = 200	ITT: yes	
		Blinding: no	All groups received EN or PN
		(10)	
El-Attar 2009 [50]	Patients with COPD	C.Random: yes	IV selenium as sodium selenite 100 μg/day, zinc 2 mg/day, and manganese 0.4 mg/day vs. none
	*n* = 80	ITT: yes	Trace elements were administered during the period on mechanical ventilation
		Blinding: yes	
		(12)	
Montoya 2009 [51]	Medical/surgical septic	C.Random: yes	Day 1 IV sodium selenite 1000 μg , day 2 sodium selenite 500 μg, and thereafter 200 μg during 7 additional days vs. selenite 100 μg/day
	ICU patients	ITT: yes	
	*n* = 68	Blinding: double	
		(7)	
Andrews 2011 [52]	Mixed ICU, multicenter	C.Random: yes	500 μg selenium supplemented PN (12.5 g nitrogen, 2000 kcal) vs. standard PN (12.5 g nitrogen, 2000 kcal) initiated after ICU admission (actual median 2.6 days) for 7 days (actual duration, mean 4.1 days).
	*n* = 502	ITT: yes	
		Blinding: double blind	
		(13)	
Manzanares 2011 [53]	Septic or trauma patients	C.Random: not sure	IV selenium supplementation loading dose 2000 μg (2 h) on day 1 followed by 1600 μg/day for 10 days vs. NaCl as placebo
	*n* = 35	ITT: no (except mortality)	
		Blinding: single blind	
		(9)	
Valenta 2011 [54]	Patients with sepsis or SIRS	C.Random: not sure	IV selenium supplementation loading dose 1000 μg on day 1 followed by 500 μg/day for 5–14 days + <75 μg/day of sodium selenite added to PN vs. NaCl + <75 μg/day of sodium selenite added to PN
	*n* = 150	ITT: yes	
		Blinding: no	
		(8)	
Heyland 2013 [16]	Multicenter mixed ICUs	C.Random: yes	500 μg selenium via PN + 300 μg selenium, 20 mg zinc, 10 mg β-carotene, 500 mg vitamin E, 1500 mg vitamin C via EN vs. placebo via PN and EN
	*n* = 1218	ITT: yes	
		Blinding: double	
		(12)	
Woth 2014 [14]	Mixed ICU, severe septic patients with multiorgan failure	C.Random: not sure	1000-μg/30 minutes loading dose of sodium selenite and 1000-μg/day treatment for a maximum of 14 days vs. control group (not described)
	*n* = 40	ITT: yes	
		Blinding: no	
		(6)	
Bloos 2016 [17]	Multicenter mixed ICU patients with severe sepsis or septic shock in last 24 h	C.Random: yes	IV loading dose of 1000 μg sodium selenite followed by continuous IV of 1000 μg sodium selenite daily until ICU discharge or for 21 days, whichever comes first vs. placebo (NaCl)
		ITT: yes	
	*n* = 1089	Blinding: double	
		(12)	
Chelkeba 2015 [15]	Mixed ICU patients with severe sepsis and septic shock	C.Random: yes	IV loading dose of 2000 μg sodium selenite followed by continuous IV of 1500 μg sodium selenite daily until day 14 vs. standard therapy without selenium
	*n* = 54	ITT: no	
		Blinding: single	
		(11)	

*Abbreviations: APACHE* Acute Physiology and Chronic Health Evaluation, *COPD* Chronic obstructive pulmonary disease, *C.Random* Concealed randomization, *D5W* Dextrose 5 % in water, *EN* Enteral nutrition, *ICU* Intensive care unit, *ITT* Intention to treat, *IV* Intravenous, *PN* Parenteral nutrition, *SIRS* Systemic inflammatory response syndrome, *TBSA* Total body surface area

**Table 2:** Reported outcomes of included randomized clinical trials evaluating intravenous selenium supplementation in critically ill patients

Study	Mortality (%)		Infections (%)		LOS days	
	Experimental	Control	Experimental	Control	Experimental	Control
Kuklinski 1991 [38]	ICU 0/8 (0)	ICU 8/9 ( 89)	NR	NR	NR	NR
Zimmerman 1997 [39]	3/20 (15)	8/20 (40)	NR	NR	NR	NR
Berger 1998 [40]	1/10 (10)	0/10 (0)	1.9 ± 0.9 (1–4)	3.1 ± 1.1 (2–5)	ICU	ICU
			per patient	per patient	30 ± 12 (10)	39 ± 13 (10)
					Hospital	Hospital
					54 ± 27 (10)	66 ± 31 (10)
Angstwurm 1999 [41]	Hospital	Hospital	NR	NR	NR	NR
	7/21 (33)	11/21 (52)				
Porter 1999 [42]	0/9 (0)	0/9 (0)	5/9 (56)	8/9 (89)	ICU	ICU
					22 ± 25.2	35.8 ± 21.9
					Hospital	Hospital
					31.3 ± 23.4	49 ± 30
Berger 2001 [43]	(a) Selenium alone	1/11 (9)	(a) Selenium alone	5/11 (42)	(a)	ICU
	2/9 (22)		5/9 (56)		ICU	8.6 ± 8.1 (12)
	(b) Selenium + zinc + α-tocopherol		(b) Selenium + zinc + α-tocopherol		8.0 ± 4.0 (9)	Hospital
					Hospital	64 ± 39 (12)
	0/11 (0)		3/11 (27)		82 ± 78 (9)	
					(b)	
					ICU	
					5.8 ± 4.4 (11)	
					Hospital	
					60 ± 48 (11)	
Linder 2004 [44]	Not specified	Not specified	NA	NA	Hospital	Hospital
	5/32 (15.6)	3/35 (8.6)			24 (9–44)	26 (11–46)
Angstwurm 2007 [45]	28-day	28-day	New infections (HAP)	New infections (HAP)	ICU	ICU
	46/116 (40)	61/122 (50)	10/116 (9)	10/122 (8)	15.1 ± 10 (116)	12.7 ± 9 (122)
Berger 2007 [46]	1/11 (9)	1/10 (10)	2.1 ± 1.0	3.6 ± 1.3	ICU	ICU
			per patient	per patient	35 ± 27 (11)	47 ± 37 (10)
Forceville 2007 [47]	28-day	28-day	Superinfection	Superinfection	ICU	ICU
	14/31 (45)	13/29 (45)	1/31 (3)	2/29 (7)	21 (7–40)	18 (10–31)
	6-month	6-month			Hospital	Hospital
	18/31 (59)	20/29 (68)			25 (7–68)	33 (11–51)
	1-year	1-year				
	66 %	71 %				
Mishra 2007 [48]	ICU 8/18 (44)	ICU 11/22 (61)	1.5 ± 1.9	1.8 ± 1.6	ICU	ICU
	Hospital	Hospital	per patient	per patient	21.3 ± 16.2 (18)	20.8 ± 21.8 (18)
	11/18 (61)	15/22 (68)				
	28-day	28-day				
	8/18 (44)	11/22 (50)				
Berger 2008 [49]	ICU	ICU	36/102 (35)	34/98 (35)	ICU	ICU
	8/102 (8)	5/98 (5)			5.8 ± 5.4 (102)	5.4 ± 5.7 (98)
	Hospital	Hospital			Hospital	Hospital
	14/102 (14)	9/98 (11)			23 ± 20 (102)	26 ± 20 (98)
	3-month	3-month				
	14/102 (14)	11/98 (11)				
El-Attar 2009 [50]	ICU	ICU	VAP	VAP	NR	NR
	2/40 (5.6)	1/40 (2.9)	5/36 (14)	7/34 (21)		
Montoya 2009 [51]	Hospital	Hospital	NR	NR	Hospital	Hospital
	6/34 (18)	8/34 (24)			12 (12–14)	17 (14–20)
Andrews 2011 [52]	ICU	ICU	Confirmed	Confirmed	ICU	ICU
	84/251 (33)	84/251 (33)	104/251 (41)	121/251 (48)	13.2 (IQR 7.8–23.7)	15.1 (IQR 8.3–28.4)
	6-month	6-month			Hospital	Hospital
	107/251 (43)	114/251 (45)			29.8 (IQR 14.7–52.4)	31.2 (IQR 15.1–57.8)
Manzanares 2011 [53]	ICU	ICU	VAP	VAP	ICU	ICU
	3/15 (20)	5/16 (31)	3/15 (20)	7/16 (44)	14 ± 11 (15)	13 ± 6 (16)
	Hospital	Hospital				
	5/15 (33)	7/16 (44)				
Valenta 2011 [54]	28-day	28-day	NR	NR	NR	NR
	19/75 (25)	24/75 (32)				
Heyland 2013 [16]	Hospital	Hospital	All	All	ICU	ICU
	216/617 (35)	199/601 (33)	168/617 (27)	181/601 (30)	14.2 ± 22.7 (617)	13.8 ± 23.1 (601)
	14-day	14-day	VAP	VAP	Hospital	Hospital
	154/617 (25)	132/601 (22)	71/617 (12)	95/601 (16)	31.2 ± 50.2 (617)	29.5 ± 44.8 (601)
	28-day	28-day				
	190/617 (31)	173/601 (29)				
	3-month	3-month				
	239	222				
	6-month	6-month				
	250	235				
Woth 2014 [14]	In 14-day study period	In 14-day study period	Gram-negative	Gram-negative	NR	NR
	9/21 (43)	11/19 (58)	8/21 (38)	3/19 (16)		
			Gram-positive	Gram-positive		
			3/21 (14)	2/19 (11)		
			Fungal	Fungal		
			1/21 (5)	0/19 (0)		
Bloos 2016 [17]	28-day	28-day	Secondary infections, day 14	Secondary infections, day 14	ICU	ICU
	152/543 (28)	137/546 (25)			11 (5–22)	12 (6–24)
	90-day	90-day	243/543 (44.7 %)	269/546 (49.3 %)	Hospital	Hospital
	198/543 (38)	201/546 (38)	Secondary infections, day 21	Secondary infections, day 21	26 (16–42)	29 (17–50)
			319/543 (58.8 %)	323/546 (59.2 %)		
Chelkeba 2015 [15]	28-day	28-day	VAP	VAP	ICU	ICU
	9/29 (31)	10/25 (40)	16/26 (61.5)	21/25 (84)	19.7 ± 11 (26)	23.8 ± 13 (25)
					Hospital	Hospital
					25.2 ± 10 (26)	24.5 ± 9 (25)

*Abbreviations: HAP* Hospital-acquired pneumonia, *ICU* Intensive care unit, *NA* Nonattributable, *NR* Nonreported, , *VAP* Ventilator-associated pneumonia

### Primary outcome: mortality

**Fig. 1:**
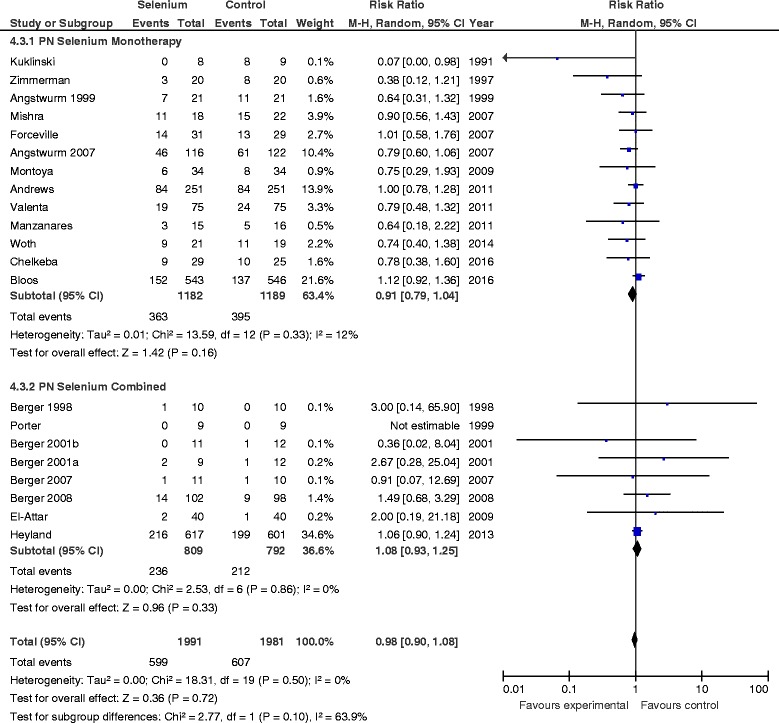
Effects of selenium therapy on mortality: subgroup analysis of monotherapy vs. combined therapy. *M-H* Mantel-Haenszel

When the results of the 21 trials in which researchers reported mortality were aggregated, no statistically significant difference was found between Se supplementation and placebo (RR 0.98, 95 % CI 0.90–1.08, *P* = 0.72, heterogeneity *I* ^2^ = 0 %) (Fig. 1). In the sensitivity analysis, after excluding the PCT guidance group of the Bloos et al. study, we found that the new RR was 0.95 (95 % CI 0.79–1.15, *P* = 0.63, *I* ^2^ = 35 %) (Additional file 1: Figure S1).

### Secondary outcomes

#### Overall effect on new infectious complications

**Fig. 2:**
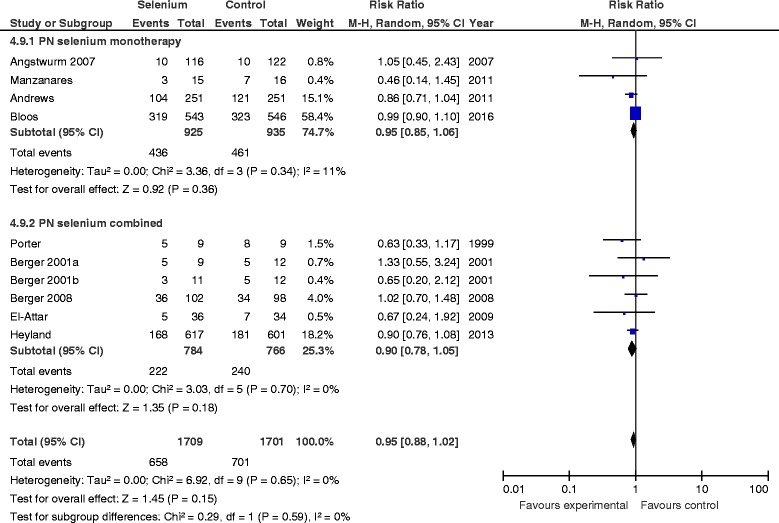
Effects of selenium therapy on infections: subgroup analysis of monotherapy vs. combined therapy. *M-H* Mantel-Haenszel

When data from ten studies were included in the meta-analysis, no significant effect of Se supplementation on infections was found (RR 0.95, 95 % CI 0.88–1.02, *P* = 0.15, *I* ^2^ = 0 %) (Fig. 2).

#### Overall effect on ICU and hospital length of stay and ventilator days

When ten RCTs in which researchers reported ICU LOS were statistically aggregated, there were no significant differences between the groups (WMD 0.32, 95 % CI −0.80 to 1.43, *P* = 0.58, *I* ^2^ = 3 %). When the eight studies in which researchers reported hospital LOS were aggregated, there were no significant differences between the groups (WMD −0.83, 95 % CI −3.71 to 2.04, *P* = 0.57, *I* ^2^ = 0 %). Also, when the eight studies in which researchers reported ventilator days were aggregated, Se supplementation was not associated with a reduction in MV days, although significant heterogeneity was present (WMD −1.75, 95 % CI −4.30 to 0.81, *P* = 0.18, *I* ^2^ = 74 %, *P* value for test of heterogeneity = 0.0004).

#### Overall effect on new renal dysfunction

After aggregating the data from eight RCTs in which researchers reported new renal dysfunction, Se therapy was not associated with a significant reduction in the incidence of renal dysfunction (RR 0.79, 95 % CI 0.57–1.08, *P* = 0.14, *I* ^2^ = 0 %).

### Subgroup analyses

#### PN selenium monotherapy vs. combined therapy

The RR associated with parenteral Se monotherapy for mortality was 0.91 (95 % CI 0.79–1.04, *P* = 0.16; *I* ^2^ = 12 %) (Fig. 1), compared with 1.08 for studies in which researchers used Se as part of combination therapy (95 % CI 0.93–1.25, *P* = 0.33). The test of subgroup differences was not significant (*P* = 0.10). There was no effect on infections with either Se monotherapy or combined therapy (test for subgroup differences, *P* = 0.59) (Fig. 2).

#### PN selenium loading dose vs. no loading dose

**Fig. 3:**
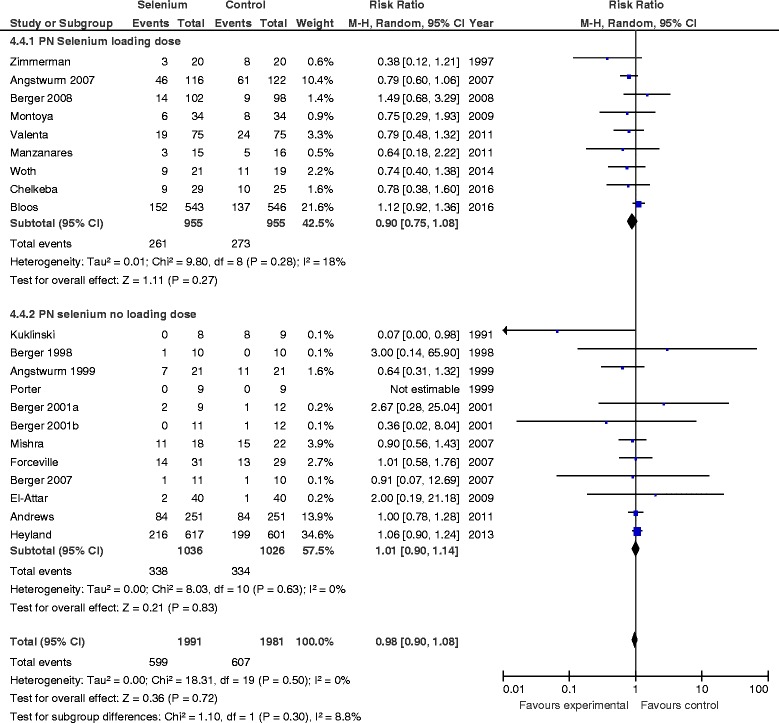
Effects of selenium therapy on mortality: subgroup analysis of loading dose vs. no loading dose. *M-H* Mantel-Haenszel

**Fig. 4:**
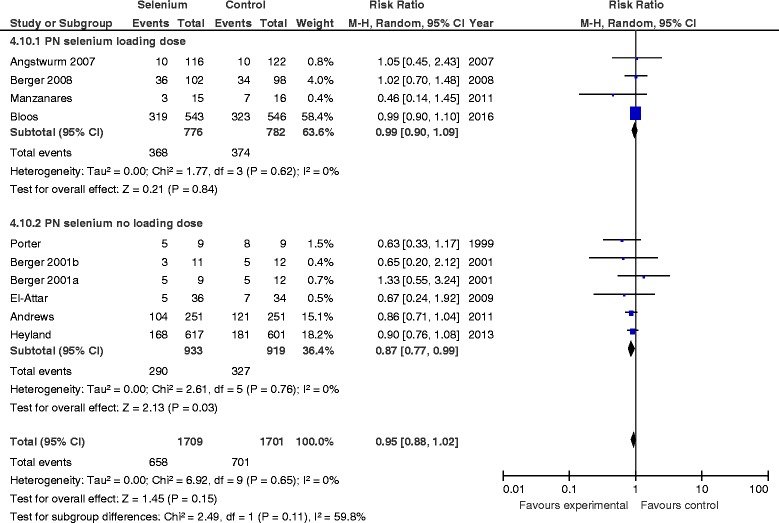
Effects of Selenium therapy on infections: subgroup analysis of loading dose vs. none. *M-H* Mantel-Haenszel

There was no treatment effect difference in mortality between studies in which researchers used a parenteral loading dose of Se as an IV bolus (RR 0.90, 95 % CI 0.75–1.08, *P* = 0.30, *I* ^2^ = 18 %) and those not using a loading dose (RR 1.01, 95 % CI 0.90–1.14, *P* = 0.83) (Fig. 3). The test for subgroup differences was not statistically significant (*P* = 0.30). There was also no effect on infectious complications in studies in which researchers used a parenteral loading dose (RR 0.99, 95 % CI 0.90–1.09, *P* = 0.84, *I* ^2^ = 0 %), whereas there was a significant reduction on infections in studies without a parenteral Se loading dose (RR 0.87, 95 % CI 0.77–0.99, *P* = 0.03, *I* ^2^ = 0 %) (Fig. 4); the test for subgroup differences was not significant (*P* = 0.11).

#### PN selenium high dose vs. low dose

There were no significant treatment effect differences in mortality (test for subgroup differences *P* = 0.89) and infections (test for subgroup differences *P* = 0.52) when we compared studies with high-dose vs. low-dose parenteral Se supplementation (data not shown).

#### Higher vs. lower mortality

The mean hospital mortality rate (or ICU mortality when hospital mortality was not reported) in the control group of all the trials was 33 %. After aggregating 11 studies with a higher mortality rate, we found that IV Se did not have an effect on mortality (RR 0.94, 95 % CI 0.84–1.05, *P* = 0.26, *I* ^2^ = 17 %). In addition, IV Se did not have an effect on mortality in eight studies with a lower mortality (RR 1.08, 95 % CI 0.90–1.08, *P* = 0.24, *I* ^2^ = 0 %). The test for subgroup differences was not significant (*P* = 0.17) (see Additional file 2: Figure S2) In addition, there were no significant treatment effect differences in infections (test for subgroup differences *P* = 0.35) when we compared studies with high vs. low mortality rates in the control group (see Additional file 2: Figure S3).

#### Study quality on outcomes

When we evaluated study quality on outcomes, a statistically significant effect of IV Se on the reduction of mortality was found in the low-quality trials (RR 0.79, 95 % CI 0.66–0.94, *P* = 0.007, *I* ^2^ = 0 %), whereas trials with higher methodology scores did not show any significant effect (RR 1.06, 95 % CI 0.95–1.19, *P* = 0.27, heterogeneity *I* ^2^ = 0 %). The overall tests for significance revealed statistically significant differences between these subgroups (*P* = 0.004) (see Additional file 3: Figure S4). Neither high- nor low-quality trials demonstrated any effect on infections (test for subgroup differences *P* = 0.72) (see Additional file 3: Figure S5).

#### Sepsis vs. nonsepsis studies

**Fig. 5:**
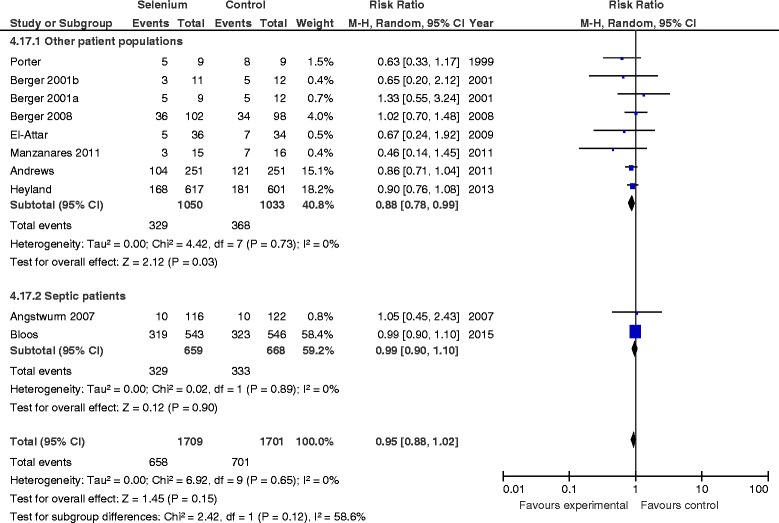
Effects of selenium therapy on infections: sepsis vs. non sepsis studies. *M-H* Mantel-Haenszel

There was no effect of Se therapy on mortality in patients with sepsis (RR 0.92, 95 % CI 0.79–1.07, *P* = 0.27, *I* ^2^ = 10 %) or without sepsis (RR 1.03, 95 % CI 0.91–1.16, *P* = 0.68; *I* ^2^ = 0 %, test for subgroup differences *P* = 0.27). However, there was a significant effect of parenteral Se on the reduction of infections in RCTs of patients without sepsis (RR 0.88, 95 % CI 0.78–0.99, *P* = 0.03, *I* ^2^ = 0 %) compared with trials of patients with sepsis (RR 0.99, 95 % CI 0.90–1.10, *P* = 0.90, *I* ^2^ = 0 %, test for subgroup differences *P* = 0.12) (Fig. 5).

#### Effect of geographic representation of study patients on outcomes

We found no significant treatment effect differences in mortality (test for subgroup differences *P* = 0.33) and infections (test for subgroup differences *P* = 0.57) when we compared studies in which researchers administered IV Se in North America vs. other geographic regions (see Additional file 4: Figs. S6 and S7).

#### Publication bias

There was no indication that publication bias influenced the observed aggregated results. In fact, funnel plots were created for each study outcome, and the tests of asymmetry showed a trend for mortality (*P* = 0.08), although the data were not significant for overall infections (*P* = 0.19), hospital LOS (*P* = 0.50), or MV days (*P* = 0.37). However, the test for asymmetry was significant for ICU LOS (*P* = 0. 047).

## Discussion

This updated systematic review and meta-analysis of the effects of parenteral Se as single or combined therapy on clinical outcomes included 21 trials with a total of 4044 critically ill patients. Including the two largest recent RCTs [16, 17], the main finding of our meta-analysis is that there was a lack of treatment effect when critically ill patients were treated with IV Se as single or combined therapy. In fact, we were unable to demonstrate any effect of IV Se supplementation on mortality or any significant effect on infections, ventilator days, or ICU and hospital LOS. Furthermore, our a priori defined subgroup analyses did not show any treatment effects on mortality. However, we found a significant effect of Se therapy on infectious complications in those studies without an initial IV loading dose, as well as a similar effect in trials conducted in nonseptic patients.

Over the last few years, different meta-analyses of Se supplementation in the critically ill have been published [8–13]. The differences with our present review are due largely to the variations in the studies included in the reviews, as our systematic review and meta-analysis is the first to include the two largest trials done to date on Se therapy in the critically ill.

Our present findings do not support the concept of pharmaconutrition by which micronutrients such as Se are provided in high (i.e., supraphysiological) doses in order to derive a pharmacological effect. Conversely to previous findings regarding IV high-dose Se monotherapy [8, 9], we did not find an overall effect of Se on infectious complications in the critically ill, although parenteral Se without an initial bolus significantly reduced infections. Nonetheless, the overall point estimate on infections of Se monotherapy is primarily and largely influenced by the Bloos et al. [17] study, the largest trial (*n* = 1089) (n= 1089) on pharmaconutrition with high-dose Se monotherapy and PCT-guided antibiotic therapy in patients with severe sepsis and septic shock. After giving an initial IV loading dose of 1000 μg sodium selenite followed by a continuous infusion of 1000 μg sodium selenite daily for no longer than 21 days, Bloos et al. [17] found that secondary infections were similar in both groups of patients. Interestingly enough, the SISPCT study [17] demonstrated that high-dose Se supplementation had no therapeutic benefit in septic patients, although plasma Se depletion at baseline was restored to the normal range already by treatment day 1, suggesting that correction of plasma Se concentration may have no beneficial value. According to this study, plasma Se levels in the Se or placebo groups were not affected by allocation to the PCT guidance or non-PCT guidance group. Our sensitivity analysis showed that, after excluding the PCT guidance arm, the new RR was 0.95 (previous RR 0.98), which confirms that excluding the PCT arm of the Bloos et al. study [17] did not affect the overall result of our analysis.

In contrast to previous knowledge, we were unable to find beneficial effects with daily doses higher than 500 μg or with providing an initial loading dose as an IV bolus (usually 1000–2000 μg in 30 minutes to 2 h). However, the absence of a significant test of subgroup differences weakens any inferences drawn from this subgroup analysis, but it likely shows the ineffectiveness of employing a loading dose with an aim of improving outcomes. So far, it has been proposed that a loading dose of 1000–2000 μg Se as pentahydrate sodium selenite has prooxidant and cytotoxic effects [1, 53, 55]. In a previous pharmacokinetic study [31], it was demonstrated that an initial IV bolus of 2000 μg followed by a continuous infusion of 1600 μg/day was the most effective dose for returning serum Se to physiologic levels and safely maximizing extracellular GPx activity and therefore the antioxidant capacity in critically ill patients. According to current knowledge, a very high Se concentration may be able to produce an inhibition of nuclear factor-κB binding to DNA, controlling gene expression and the synthesis of proinflammatory cytokines [56, 57], and also may be able to induce apoptosis and cytotoxicity in activated proinflammatory cells [58]. In addition, using an experimental model of sepsis, Wang et al. [59] demonstrated that an IV Se bolus improved hemodynamic status, decreased inflammation biomarkers, and reduced mortality. Meanwhile, contrary to our present data, in 2012 we found that a parenteral loading dose showed a trend toward reduction in mortality, whereas studies that did not use a bolus-loading dose did not show any effect on mortality. Similarly, Huang et al. [9], after aggregating nine RCTs on Se monotherapy, demonstrated that an IV bolus was associated with a significant reduction in mortality (RR 0.73, 95 % CI 0.58–0.94, *P* = 0.01). However, neither of the previous meta-analyses considered the SISPCT study [17].

So far, most clinical studies using Se at low doses have been underpowered and have involved Se administered in a cocktail approach. Thus, positive results in those trials cannot be clearly attributed solely to Se supplementation. Notwithstanding this, according to the concept of nutrient replacement, by which micronutrient substitution is aimed at replenishing losses and target restoration of physiological function [60], Se must be supplemented at standard doses by the enteral (77–100 μg/day) or the parenteral (100–400 μg/day) route [61] because the results of our meta-analysis do not refute previously recommended Se substitution doses.

Despite earlier results of the Angstwurm study [41], which demonstrated that IV high-dose Se substitution in septic patients with an Acute Physiology and Chronic Health Evaluation II score higher than 15 significantly reduced the requirements of renal replacement therapy (*P* = 0.035), the post hoc analysis of the REDOXS [16] study demonstrated that patients with renal failure might have a worse outcome when treated with high-dose antioxidants. In fact, Heyland and coworkers [16] demonstrated that both glutamine and antioxidants appeared harmful in patients with baseline renal dysfunction, showing a higher 28-day mortality (OR 3.39, 95 % CI 1.41–8.17, and 3.07, 95 % CI 1.24–7.59, for antioxidants alone and glutamine plus antioxidants, respectively). Nonetheless, in the recently published SISPCT study [17], researchers did not find any risk of increased harm in patients with baseline renal failure. In addition, in the present study, we were unable to find any deleterious effect of Se therapy on renal function in the critically ill.

Thus, the current understanding of why there is a lack of therapeutic effect of IV Se therapy in critically ill patients and patients with severe sepsis remains unclear. Notwithstanding this, Se therapy could show benefits in other patient populations that were not considered in our meta-analysis. In fact, it is currently known that circulating Se levels significantly decrease in the perioperative period of cardiac surgery [62]. Also, in a nonrandomized interventional trial, Stoppe et al. [63] demonstrated that high-dose sodium selenite therapy as a pharmaconutrient strategy was effective in preventing the decrease of Se levels and that clinical outcomes may be superior in supplemented patients compared with a historical control group. The SodiUm SeleniTe Administration IN Cardiac Surgery (SUSTAIN CSX®-trial, ClinicalTrials.gov identifier NCT02002247), an RCT aimed at evaluating the effects of perioperative high-dose Se supplementation in high-risk cardiac surgical patients undergoing complicated open heart surgery, is currently recruiting participants [64].

According to current evidence derived from recent trials and our meta-analysis, the updated version of Canadian Clinical Guidelines [65] recommended not using IV Se alone or in combination with other antioxidants in critically ill patients, which means that this strategy has recently been downgraded.

The strength of our meta-analysis is based on the fact that we used several methods to reduce bias (comprehensive literature search, duplicate data abstraction, comprehensive search strategy using specific criteria, and including non-English-language articles), we contacted trial authors to obtain additional data and refine our analysis, and we ultimately focused on clinically important primary outcomes in ICU patients. In addition, given the wide variety of clinical diagnoses and the heterogeneous population of ICU patients included in this systematic review (sepsis, septic shock, trauma, pancreatitis, surgical ICU patients), the results and conclusions may be applied to a broad and heterogeneous group of critically ill patients. While having a fairly large overall sample size and low heterogeneity, which makes the estimate quite robust, our subgroup analyses are limited by the small number of trials.

## Conclusions

In this updated systematic review and meta-analysis, we found that parenteral Se as single or combined therapy with other antioxidant micronutrients had no effect on mortality, infections, renal function, ICU and hospital LOS, or ventilator days. Moreover, subgroup analyses did not show any treatment effects on mortality, although a significant effect on infections was found in those studies performed in nonseptic patients and when high-dose Se as an initial IV bolus was not administered.

According to our findings, IV Se therapy cannot be recommended for routine clinical use in critically ill patients. In view of these results, we suggest that we need to go back to basics and obtain more pharmacokinetic and pharmacodynamic data in specific patient populations with specific dosing strategies. We strongly believe that, without this first step exploring pharmacokinetic data, no further research on parenteral Se monotherapy in critically ill patients is warranted.

## Key messages

- Se is an essential trace element with antioxidant, immunomodulatory, and anti-inflammatory effects that has been considered the cornerstone of the antioxidant defense system.
- Recently, in the largest trial on IV Se monotherapy, investigators were unable to find any clinical benefit of high-dose sodium selenite in patients with sepsis and septic shock.
- According to our findings, there is no evidence for a beneficial effect on mortality, infections, and other relevant clinical outcomes of high-dose IV Se as single or combined therapy (antioxidant cocktails) in critically ill patients.
- IV Se may be able to significantly reduce infections in those studies performed with nonseptic patients and when high-dose Se as an initial IV bolus is not administered.

## Additional files

Additional file 1:
**Figure S1.** Effect of Se on mortality. Sensitivity analysis after excluding the PCT guidance group in the SISPCT study. (DOCX 82 kb)
Additional file 2:
**Figure S2.** Effect of IV Se on mortality: high- vs. low-mortality trials. **Figure S3.** Effect of IV Se on infections: high- vs. low-mortality trials. (DOCX 189 kb)
Additional file 3:
**Figure S4.** Effect of IV Se on mortality: higher- vs. lower-quality trials. **Figure S5.** Effect of IV Se on infections: higher- vs. lower-quality trials. (DOCX 188 kb)
Additional file 4:
**Figure S6.** Effect of geographic representation of study patients on outcomes: mortality. **Figure S7.** Effect of geographic representation of study patients on outcomes: infections. (DOCX 190 kb)

## Abbreviations

APACHE: Acute Physiology and Chronic Health Evaluation
C.Random: Concealed randomization
COPD: Chronic obstructive pulmonary disease
D5W: Dextrose 5 % in water
EN: Enteral nutrition
GPx: Glutathione peroxidase
HAP: Hospital-acquired pneumonia
Hosp: Hospital
ICU: Intensive care unit
ITT: Intention to treat
IV: Intravenous
LOS: Length of stay
M-H: Mantel-Haenszel
MV: Mechanical ventilation
NA: Nonattributable
NR: Nonreported
PN: Parenteral nutrition
RCT: Randomized clinical trial
REDOXS: REducing Deaths due to Oxidative Stress trial
RevMan: Review Manager software
Se: Selenium
SIRS: Systemic inflammatory response syndrome
SISPCT: Sodium Selenite and Procalcitonin Guided Antimicrobial Therapy in Severe Sepsis study
SUSTAIN CSX®: SodiUm SeleniTe Administration IN Cardiac Surgery trial
TBSA: Total body surface area
VAP: Ventilator-associated pneumonia
WMD: Weighted mean difference
