# Supplementary material for: High-dose intravenous selenium does not improve clinical outcomes in the critically ill: a systematic review and meta-analysis
Source: Crit Care. 2016 Oct 28;20:356. doi: 10.1186/s13054-016-1529-5 (PMC5084353; doi:10.1186/s13054-016-1529-5)
Supplement: Additional file 4: — Figure S6. Effect of geographic representation of study patients on outcomes: mortality. Figure S7. Effect of geographic representation of study patients on outcomes: infections. (DOCX 190 kb) [file 13054_2016_1529_MOESM4_ESM.docx]

**Intravenous High-dose Selenium Does Not Improve Clinical Outcomes in the Critically Ill: A Systematic Review and Meta-analysis**

**William Manzanares, Margot Lemieux, Gunnar Elke, Pascal L. Langlois,**

**Frank Bloos, Daren K. Heyland**

**Additional File 4**

**
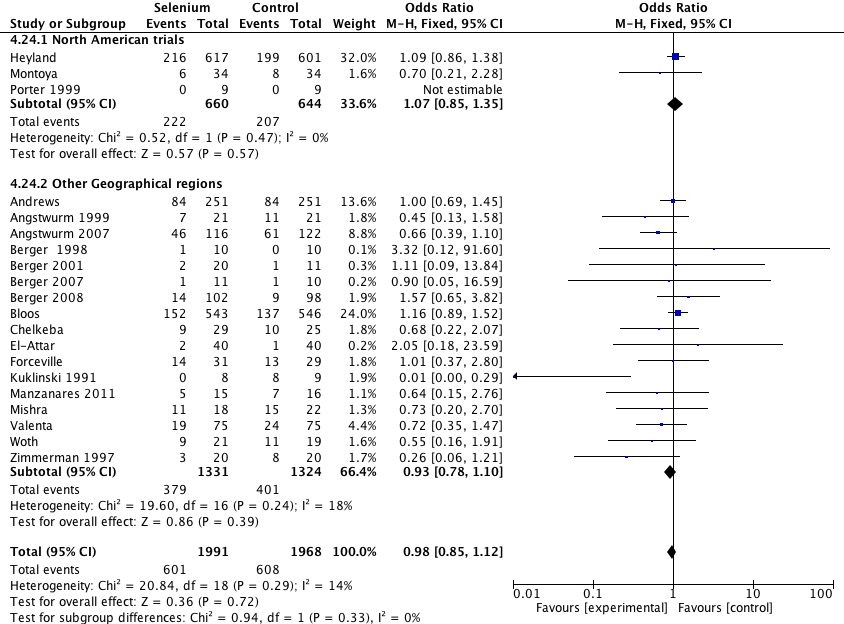
**

**Figure S6 :** Effect of geographic representation of study patients on outcomes: Mortality.

CI, confidence interval; M-H, Mantel-Haenszel test.

**
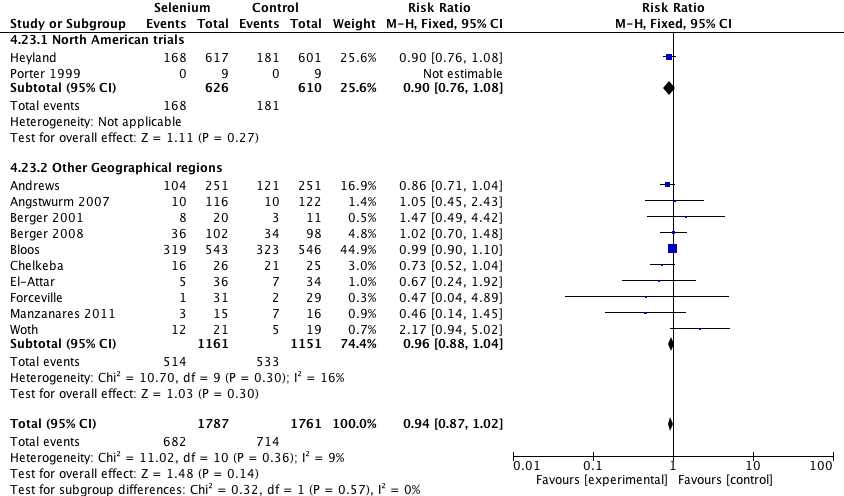
**

**Figure S7 :** Effect of geographic representation of study patients on outcomes: Infections.

CI, confidence interval; M-H, Mantel-Haenszel test.
